# The role and therapeutic implications of T cells in cancer of the lung

**DOI:** 10.1002/cti2.1076

**Published:** 2019-08-28

**Authors:** Samuel C Neeve, Bruce WS Robinson, Vanessa S Fear

**Affiliations:** ^1^ National Centre for Asbestos Related Diseases (NCARD) Lv5 QQ Block (M503) QEII Medical Centre The University of Western Australia Perth WA Australia; ^2^ School of Biomedical Sciences The University of Western Australia Perth WA Australia; ^3^ Centre for Respiratory Health The University of Western Australia Perth WA Australia; ^4^ Telethon Kids Institute Perth WA Australia

**Keywords:** immunotherapy, lung cancer, T cells

## Abstract

Lung cancer remains the leading cause of cancer‐related death worldwide. The disease is classified into two major subtypes, small‐cell lung cancer (SCLC) and the more prevalent non‐small‐cell lung cancer (NSCLC). First‐line conventional therapies, such as chemotherapy, radiotherapy and surgery, have offered limited benefit, and patient prognosis remains poor with post‐treatment recurrences representing a major cause of morbidity. Consequently, there is an urgent need for improved therapeutic options. Historically, NSCLC has been considered a non‐immunogenic disease. However, increased understanding of tumor‐immune interactions has challenged this paradigm in both lung and other malignancies, with cancer elimination by tumor‐specific T cells increasingly well described in a myriad of solid tumors. Recent evidence has demonstrated that absent or weak anticancer responses are likely a product of tumor‐derived immunosuppression. This knowledge, along with a greater appreciation for the role of T cells in lung cancer elimination, has driven development of novel immunotherapeutic approaches which are demonstrating remarkable clinical efficacy. This review examines the role of T cells in lung cancer, discussing the direction and clinical significance of current and future immunotherapeutic strategies.

## Introduction

Lung cancer is a highly invasive and metastatic disease representing the fifth most commonly diagnosed cancer in Australia and the leading cause of cancer‐related deaths worldwide.^1^ The disease is classified into two broad histopathological subtypes, small‐cell lung cancer (SCLC) and, the predominant form, non‐small‐cell lung cancer (NSCLC).^2^ Following diagnosis, lung cancer is staged using the tumor nodal metastasis system, which assesses primary tumor size, lymph node (LN) involvement and metastasis.^3^ Depending on disease stage, treatment generally consists of standard therapeutic regimes including surgery, chemotherapy and radiotherapy.^2^

During early stages of disease, surgery remains the primary curative treatment option, often consisting of a complete lobectomy in conjunction with widespread LN resection for staging purposes.^4^ The current 8^th^ edition TNM system recommends removal of six nodes from six LN stations, regardless of tumor burden.^3^ In later stages, surgery is typically combined with adjuvant platinum‐based and cytotoxic chemotherapies, and/or radiotherapy.^2^ However, despite these treatment options, patient prognosis remains poor, with a 5‐year survival for all stages combined of 16.6%.^2^ This is largely due to presurgery metastatic disease or development of microscopic residual disease. Hence, there is a need for improved treatment options for lung cancer patients.

Historically, many solid tumors, including lung cancer, have been considered relatively non‐immunogenic diseases.^5^ However, recent research has challenged this paradigm, demonstrating the immunogenic nature of lung cancer and highlighting the ability of tumors to suppress the immune response within the tumor microenvironment (TME).^2^, ^5^ This increased understanding has driven the development of various types of immunotherapies which look to overcome this suppression and unleash pre‐existing immune responses that target the tumor. In the past few years, immunotherapies, such as monoclonal antibodies, adoptive cell transfers (ACTs) and vaccines, have become increasingly integrated into the clinic for treatment of various types of cancers, such as melanoma, and, more recently, for lung cancer.^2^ Within the last decade, the discovery of antibodies that target the immune checkpoints, programmed cell death 1 (PD‐1) and programmed death‐ligand 1 (PD‐L1) has revolutionised the treatment of NSCLC.^5^ This review discusses the complex nature of the immune cell response in tumor development and more specifically focuses on the role of T cells in new immunotherapeutic strategies for the treatment of lung cancer.

## Immune cell recognition of tumor cells in lung cancer

The immune system is critical in recognising and eliminating cancer cells.^6^ In NSCLC, T‐cell responses have been noted against lung cancer tumor‐associated antigens (TAAs), such as cancer–testis antigens (e.g. MAGE‐A3).^2^ In addition, tumor neoantigens, which are antigens expressed exclusively on tumor cells, have also been shown to initiate antitumor immune responses.^7^ However, despite T‐cell recognition, tumors still develop due to direct tumor immunoediting, immune cell suppression and/or an inhibitory cytokine milieu.^8^

Immunoediting describes how continual selective pressure exerted on immune‐recognised tumor cells can shape a more resistant tumor cell population, leading to outgrowth of tumor cells that can escape immunosurveillance or inhibit immune cell activity.^8^ Recent studies have determined that ongoing selective pressures from tumour‐immune cell response in early‐stage NSCLC drive MHC dysregulation, disrupted antigen presentation and depleted neoantigen expression, all of which correlated to poorer disease‐free survival.^9^, ^10^ Furthermore, the growing tumor develops an immunosuppressive TME, characterised by increased immunosuppressive cytokines and immune cells, thereby further potentiating tumor growth.^11^

In effect, a T‐cell response to lung tumor cells does not ensure an immune system antitumor response.^8^ Accordingly, an understanding of the tumor‐immune relationship has become a primary research focus in clinical oncology. This is particularly important in the wake of recent clinical approval, by the Therapeutic Goods Administration, for treatment of NSCLC patients with nivolumab (2018) and pembrolizumab (2019). Whilst these immunotherapy treatments have improved patient outcomes, a significant proportion of patients do not respond to treatment. An understanding of the underlying immune cell response to these treatments will guide future directions in optimisation of current immunotherapy. This knowledge will be vital in determining effective combination immunotherapy regimes and the development of future immune checkpoint therapeutic antibodies.

## The tumour‐immune cell response in lung cancer

**Figure 1:**
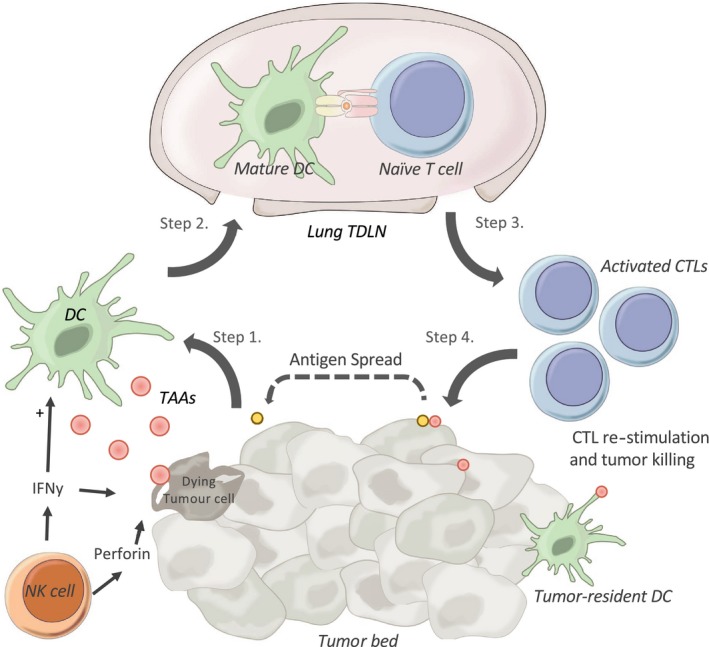
A functional cytotoxic tumour‐immune cell response. Tumor‐associated antigens (TAAs) are collected by peripheral dendritic cells (DCs), which traffic to tumor‐draining lymph nodes (TDLNs), where they present TAAs to naïve T cells, thereby activating them. These naïve T cells can expand into clonal populations of cytotoxic T lymphocytes (CTLs), which migrate to the tumor site. Here, they recognise their cognate antigen on either tumor cells directly or are re‐stimulated by tumor‐resident DCs, which initiates a cytotoxic response directed at the tumor cells. Tumor cell death may release additional TAAs, which can prime new immune responses, in a process known as antigen spread. NK cells, natural killer cells.

The tumour‐immune cell response is thought to commence with the recognition of TAAs or neoantigens that are either released into the TME or recognised on the surface of tumor cells themselves (Figure 1).^7^, ^8^ Inflammatory signals induced by growing tumors may recruit infiltrative innate cells, such as NK cells, to the tumor site, which induce tumor cell apoptosis through interferon gamma (IFNγ) and perforin release. 8 These apoptosing tumor cells release tumor debris and damage‐associated molecular patterns, which further escalate inflammatory cytokine and chemokine secretion, recruiting antigen‐presenting cells (APC), such as dendritic cells (DCs), into the TME.^12^ DCs capture tumor antigen and migrate to lung tumor‐draining lymph nodes (TDLNs), where they present TAAs on major histocompatibility complex (MHC) molecules to naïve T cells, triggering activation of TAA‐specific CD4^+^ helper T cells (steps 1–2), or to CD8^+^ cytotoxic T cells (CTLs) (step 3), via MHC class II or MHC class 1, respectively.^2^, ^8^ The activated CTLs then migrate back to the tumor, in response to chemokine signalling (step 3). Within the TME, CTLs are re‐stimulated by their cognate antigen on tumor‐resident APC or directly on MHC class I on tumor cells (step 4), leading to tumor cell killing and potential spread of neoantigen responses which may prime secondary immune responses.^6^ However, given that lung tumor growth progresses, these tumor‐infiltrating lymphocytes (TILs) must fail to effectively eliminate tumor cells.^6^, ^8^

## TILs in lung cancer

**Figure 2:**
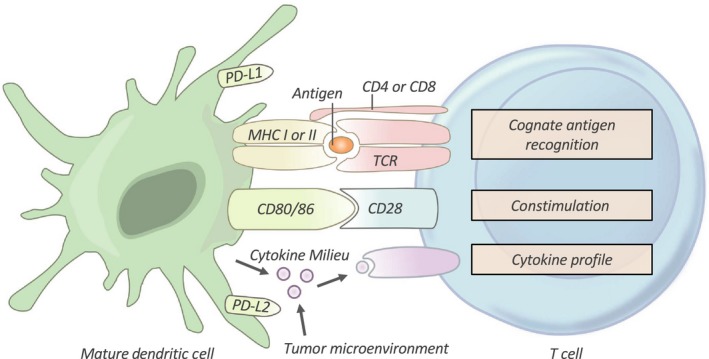
T‐cell Activation. T cells require three signals from an antigen‐presenting cell (APC) in order to be effectively activated and primed. First, T cells must recognise their cognate antigen in the context of the correct major histocompatibility complex (MHC). Second, T cells require appropriate costimulation by an APC. Finally, the T cell receives instructive cytokines from immune cells and the tumor microenvironment which dictate its phenotypic differentiation. TCR, T‐cell receptor; PD‐L1/PD‐L2, programmed death‐ligands 1 and 2.

Tumor‐infiltrating lymphocytes are a heterogeneous population comprised of CD8^+^ cytotoxic T cells, CD4^+^ helper T cells and FOXP3^+^ regulatory T cells (Tregs).^13^ Activation of TILs is highly regulated, requiring recognition of antigen in the context of appropriate MHC molecules (Figure 2) in conjunction with ligation of their costimulatory molecules such as CD28, CD40, OX40, GITR, 4‐1BB and ICOS.^5^, ^6^ Conversely, engagement of coinhibitory receptors (immune checkpoints) such as programmed death‐ligand 1 (PD‐1), cytotoxic T‐lymphocyte antigen‐4 (CTLA‐4) and lymphocyte activation gene‐3 (LAG‐3) provides negative regulation, mitigating excessive T‐cell activation.^5^, ^14^ These coinhibitory receptors are often transiently expressed post‐activation, but in the context of chronic antigen exposure, such as in cancer, they may have sustained expression, thus promoting TIL dysregulation.^15^ In addition, various environmental cytokines, such as interleukin‐2 (IL‐2), IL‐15, IL‐17, IL‐10, and transforming growth factor β (TGFβ), provide a further intrinsic layer of positive and negative feedback that modulates T‐cell effector phenotype, functional quality and the antitumor immune cell response.^14^, ^16^

The correlation between TIL and CD8 T‐cell frequency and clinical outcome has been extensively studied in lung cancer, with research indicating that a greater density of TILs correlates with improved progression‐free survival.^17^ Moreover, a T helper type 1 (Th1) cytokine profile that promotes CD8 T‐cell activation also correlates with a stronger antitumor immune response in lung cancer.^12^ However, recent research has observed many late‐stage lung cancers to be characterised by consistent TIL hypofunction, indicative of an immune response that is dysfunctional and weak.^18^ Importantly, studies indicate that this suppressed antitumor immune cell response in lung cancer may be overcome by enhancing the activation status of antitumor immune cells, releasing immune‐suppressive checkpoints or increasing T‐cell frequency.^2^, ^6^

## Role of specific immune cells in the antitumor immune response to lung cancer

**Figure 3:**
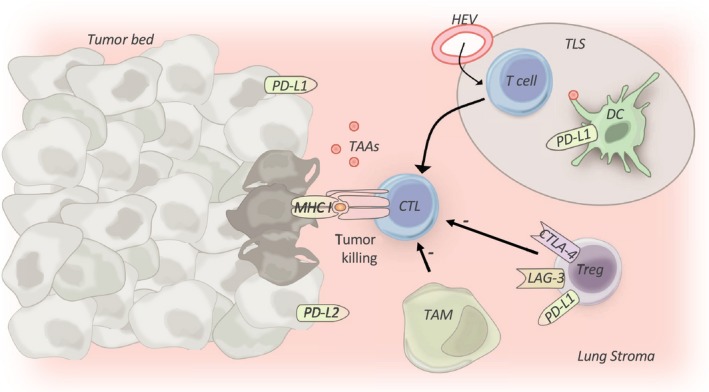
Lung cancer immune contexture**.** In lung cancer, the immune contexture surrounding the tumor is highly organised, consisting of T cells and dendritic cells (DCs) found within lymph nodes and tertiary lymphoid structures (TLSs). High endothelial venules (HEV) on TLS enable efficient migration of peripheral immune cells into the tumor microenvironment. Increased numbers of these antitumor cells, such as cytotoxic T lymphocytes (CTLs), are associated with improved prognosis, whereas increased density of protumor cells, such as tumor‐associated macrophages (TAMs) and regulatory T cells (Tregs), and increased coinhibitory molecule expression, is associated with poorer prognosis. PD‐L1/PD‐L2, Programmed death‐ligands 1 and 2; CTLA‐4, cytotoxic T‐lymphocyte antigen‐4; LAG‐3, lymphocyte activation gene 3; MHC I, major histocompatibility complex I.

In lung cancer, the immune contexture within the TME is highly diverse and is associated with clinical outcomes (Figure 3). Within the lung tumour microenvironment many cells modulate the anti‐tumour CTL response including DCs, CD4 helper T cells, CD4 T regulatory (T reg) cells, tumor‐associated macrophages (TAMs) and myeloid‐derived suppressor cells (MDSCs).^13^ These cells will be discussed in detail in this section.

### Antigen‐presenting cells

Given that many TAAs are products of tumor cell death, they are often exogenous molecules that are typically presented on APCs in the context of MHC class II, which is not directly recognised by CD8^+^ T cells.^6^, ^14^ Further complicating this, lung cancer cells are observed to downregulate MHC class I expression to avoid direct CD8^+^ T‐cell antigen recognition.^10^ Therefore, the capacity for specialised DCs to transfer these exogenous antigens bound for MHC class II expression onto MHC class I molecules in a process known as cross‐presentation is critical for priming CD8^+^ T‐cell antitumor responses.^14^ In order for effective cross‐presentation, DCs must be functionally mature, which requires both a favourable cytokine environment and DC licensing by CD4^+^ T cells through the CD40‐CD40L axis.^19^

In murine models adoptive transfer tumour‐specific CTL leads to tumour eradication in WT mice, whereas transfer of CTL to CD40 deficient mice did not lead to an effective anti‐tumour immune response.^20^ Human studies in NSCLC have observed specialised cross‐presenting DC subsets to be blocked at immature developmental stages, thus promoting cross‐tolerance, rather than cross‐priming, of CD8^+^ T cells.^21^ Furthermore, other studies have indicated that lung tumor‐infiltrating CD11b^+^ DCs strongly overexpress PD‐L1, contributing to immunosuppression and tumor growth.^22^

Tertiary lymphoid structures (TLSs) or the local TDLN provides sites for tumor antigen cross‐priming by DCs in an environment sheltered from tumor‐derived inhibitory molecules and cytokines (Figure 3).^13^ Recent research has indicated that TLSs are critical in shaping the antitumor immune response, favouring a Th1 cell phenotype.^12^ As such, in NSCLC, increased intratumoral TLS density has been observed to be associated with a greater frequency of CTLs and effector–memory TIL, and to correlate to improved patient survival.^13^

### CD4 T cells

CD4

#### T helper cells

The functional role of CD4^+^ helper T cells in the tumour‐immune cell response is less understood compared to that of CD8^+^ T cells. Despite this, emerging evidence has suggested that in lung cancer, these CD4^+^ T cells are prognostically significant, with increased tumor‐infiltrating CD4^+^ T cells correlated to improved survival.^17^ Importantly, CD4^+^ T cells are considered central to licensing DCs through CD40L signalling, enabling CD8^+^ T‐cell cross‐priming and stimulating CD8^+^ T‐cell memory development.^19^ Furthermore, recent research has demonstrated CD4^+^ T cells to be important in instigating recognition of neoantigens and driver mutations in human NSCLC tumors, with endogenous responses demonstrated in patients.^23^

#### CD4^+^ Regulatory T cells (Tregs)

Regulatory T cells (Treg) are an immunosuppressive subset of CD4^+^ T cells that express the transcription factor, FOXP3. In lung cancer, Tregs suppress antitumor CD4^+^ and CD8^+^ T‐cell responses, contributing to disease progression.^11^ Antigen recognition in the presence of transforming growth factor β (TGFβ) and IL‐10, which are produced within the lung tumor environment, induces CD4^+^ T‐cell differentiation into inducible Tregs (iTregs).^13^, ^14^ Once activated, Tregs can then exert their immunosuppressive function on effector CD4^+^ and CD8^+^ T cells, namely through further secretion of inhibitory cytokines, including TGFβ, IL‐10 and IL‐35.^11^

In NSCLC, increased levels of intratumoral and peripheral Tregs correlate with poorer prognosis and increased metastatic risk.^15^ This is further corroborated by studies in NSCLC demonstrating an increased density of CD4^+^ and CD8^+^ T cells to Tregs to correlate to improved survival.^13^, ^15^ Interestingly, Wei and colleagues demonstrated that Tregs infiltrating into NSCLC tumors have a greater density of inhibitory molecule expression, such as CTLA‐4, PD‐1 and LAG‐3, compared to peritumoral Tregs.^24^ Therefore, this may render tumor‐associated Tregs sensitive to immunotherapies that target these inhibitory molecules, such as immune checkpoint blockade therapy (ICPB).

#### Cytotoxic T lymphocytes

CD8^+^ T cells or CTLs have been demonstrated to be critical in lung cancer immunity in both preclinical models and humans.^5^, ^17^ Despite this, research indicates these cells may be functionally tolerant to tumor antigen in lung cancer, characterised by dysregulated cytotoxic function and exhaustion.^25^ Studies have demonstrated CD8^+^ TILs from lung cancer patients have increased expression of inhibitory receptors, such as PD‐1, CTLA‐4 and LAG‐3 which correlated to increased risk of disease progression,^25^ whilst others have reported CD8^+^ T cells from NSCLC patients to have reduced IFNγ production compared to healthy controls.^15^

In humans, increased CD8^+^ T cells infiltrating the tumor stroma of NSCLC are a positive prognostic marker, correlating to reduced metastatic risk.^17^ More recently, resident memory CD8 T‐cell (CD103^+^) frequency in early‐stage NSCLC correlated with improved prognosis, putatively due to their greater capacity to produce IFNγ than other TIL subsets.^26^, ^27^ Accordingly, current immunotherapy strategies are focused on increasing the induction of tumor‐specific effector CD8^+^ T cells, namely immune checkpoint blockade therapy (ICPB), ACT and antitumor vaccination.^5^

### Other leucocytes associated with tumor‐derived immunosuppression

Lung tumor cells promote an immunosuppressive TME through production of cytokines, including IL‐10, vascular endothelial growth factor and TGFβ.^11^, ^16^ In addition to recruitment and differentiation of immunosuppressive Tregs, these factors also attract TAMs and MDSCs.^11^, ^14^ Studies in NSCLC patients observed reduced numbers of immunosuppessive TAMs correlated with improved survival and reduced metastatic disease,^28^ whereas elevated levels of TAMs and MDSCs in the TME correlated with poor patient prognosis.^29^ MDSC and TAMs are reviewed elsewhere and will not be the focus of this review.^29^

## Immune checkpoint molecules in lung cancer

**Figure 4:**
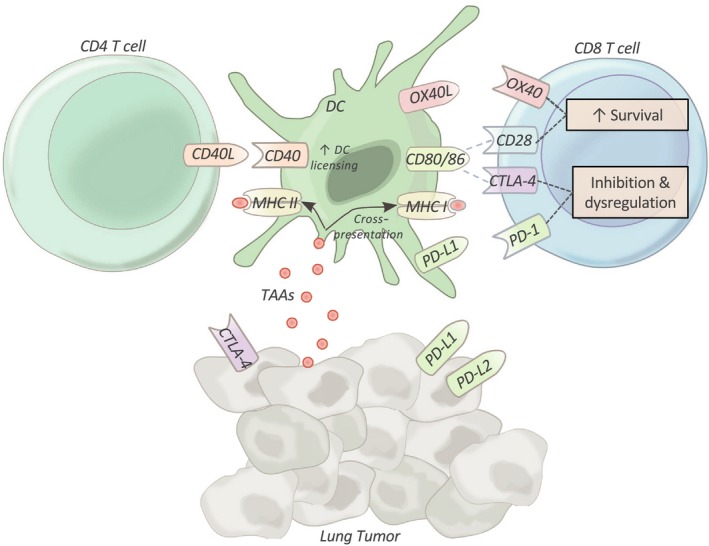
Lung cancer modulation of T‐cell responses. Lung tumor cells have the capacity to modulate immune responses in order to facilitate their escape. This includes altering the expression and interaction of costimulatory molecules, such as OX40/OX40L and CD40/CD40L, and coinhibitory molecules, such as the cytotoxic T‐lymphocyte antigen 4 (CTLA‐4) and programmed cell death 1 (PD‐1) axis. By altering these checkpoint pathways, the tumor can reduce T‐cell responses by abrogating their survival and causing dysregulation. Programmed death‐ligands 1 and 2, PD‐L1/PD‐L2; cytotoxic T‐lymphocyte antigen‐4, CTLA‐4; major histocompatibility complexes I and II, MHC I/MHC II.

Even if T cells can be activated within these immunosuppressive TMEs, lung tumor cells can exploit the homeostatic balance between costimulatory and coinhibitory signals, collectively referred to as coregulation.^6^ Costimulatory molecules such as CD28 and OX40 promote proliferation and survival.^14^ Conversely, coinhibitory molecules (immune checkpoints), such as CTLA‐4 and PD‐1, can induce T‐cell senescence and inactivation (Figure 4).^14^ Lung cancer cells can co‐opt these checkpoint pathways, with dysregulation to both the CTLA‐4 and PD‐1 axis well documented in lung cancer.^24^

### Cytotoxic T‐lymphocyte antigen 4

Cytotoxic T‐lymphocyte antigen 4 is a protein receptor expressed on T cells soon after their activation, functioning as a key negative regulator of their function. As a homologue of the costimulatory molecule, CD28, it binds both ligands CD80 and CD86 on APCs with greater affinity and avidity, blocking costimulatory signalling and transmitting an inhibitory signal to the T cell.^30^ Additionally, the literature suggests CTLA‐4 ligation may also antagonise T‐cell activation by reducing T‐cell IL‐2 responsiveness and disrupting cell cycle regulating molecules.^30^ CTLA‐4 is also expressed constitutively on Tregs, enhancing their immunosuppressive capacity.^31^

In lung cancer, patients have been observed to have greater T‐cell and Treg CTLA‐4 molecule surface density compared to intracellular density in healthy controls.^31^, ^32^ Further, studies have reported 40% of NSCLC patients to express CTLA‐4 on their tumor cells; notably increased CTLA‐4 expression in tumour tissue associated with improved survival, whereas in the TDLNs elevated CTLA‐4 expression correlated with poor prognosis.^32^ As such, therapeutically inhibiting the CTLA‐4 axis leads to increased activation of the immune system and is of clinical interest for the treatment of lung cancer.

### Programmed cell death 1

Programmed cell death 1 is an immune checkpoint in the same family as CTLA‐4. Expression of PD‐1 on activated T cells occurs later in their effector phase compared to CTLA‐4, meaning PD‐1 inhibitory regulation occurs later in the immune response.^2^, ^25^ Engagement of PD‐1 by either of its ligands, PD‐L1 or PD‐L2 expressed on tumor cells, DCs or NK cells, suppresses T‐cell activity by abrogating TCR signalling, prompting an exhaustive phenotype.^33^ Given its delayed role, PD‐1 is thought to be more significant within the TME and therefore an important mechanism of tumor‐immune resistance.

In NSCLC, intratumoral CD8^+^ T cells are found to express greater PD‐1 than peripheral populations, with this expression positively correlated to nodal status and tumor stage.^18^, ^25^ Further, others have reported that 20–60% of NSCLC tumors were positive for PD‐L1 and PD‐L2, with overexpression associated unfavourable patient prognosis.^34^ Therefore, given the role and clinical significance of the PD‐1 axis in CTL dysfunction in NSCLC, it is unsurprising that immune checkpoint blockade therapy targeting this pathway has revolutionised lung cancer therapy, providing unprecedented survival benefit for many patients.^2^, ^5^

## Current lung tumor immunotherapy

Given the strong evidence supporting the role of the immune system in the development of lung cancer, much research focus has been on the development of various immunotherapies for the disease, especially therapies that enhance T‐cell‐mediated responses. Within the last decade, significant advances have been made in lung cancer immunotherapy, with immune checkpoint blockade therapy (ICPB) targeting CTLA‐4 and PD‐1 representing the most widely studied immunotherapies at present.^2^ The early success of anti‐CTLA‐4 (aCTLA‐4) and anti‐PD‐1 (aPD‐1) treatment drove further research efforts to exploit the immune cell response in cancer therapy, which has led to the clinical integration of other immunotherapies, such as adoptive cell therapy and neoantigen vaccination. This section will discuss current clinical findings relevant to immunotherapy treatment in lung cancer patients.

### aCTLA‐4

Given the role of CTLA‐4 in immune tolerance and Treg‐mediated suppression, CTLA‐4 blockade (aCTLA‐4) using monoclonal antibodies has been extensively studied as a potential ICPB therapy.^2^ Preclinical murine studies demonstrated aCTLA‐4 to augment antitumor responses, triggering regression in models of ovarian, bladder and brain tumors.^35^ Early murine research indicated the efficacy of aCTLA‐4 to function through Treg depletion, although recent literature challenged this, demonstrating that in human cancers, this may not occur.^36^ As such, aCTLA‐4 efficacy may rely on antagonism of the competitive inhibition of TIL costimulation to enhance T‐cell activation or via other unelucidated mechanisms.

Demonstration of preclinical efficacy of CTLA‐4 antibodies prompted clinical trials, with a phase‐III trial observing improvement in overall survival for patients with advanced melanoma. This trial established significant survival advantages with a median overall survival rate of 10.0 months on ipilimumab, an IgG1 antibody targeting CTLA‐4, compared to 6.4 months in patients receiving gp100 peptide vaccine alone.^37^ These findings led to US Food and Drug Administration (FDA) approval of ipilimumab as a first‐line therapy for advanced melanoma in 2011. In lung cancer, ipilimumab has demonstrated poor efficacy in clinical studies.^2^, ^5^ Recently, a phase‐II trial investigating tremelimumab, an IgG2 antibody targeting CTLA‐4, in patients with relapsed malignant mesothelioma found no significant improvement to survival compared to placebo.^38^

### aPD‐1

PD‐1 blockade (aPD‐1) is thought to operate by restoring immunosuppressed CD8^+^ TIL effector functions and enhancing their cytotoxic activity against tumor cells by blocking the binding of PD‐1 to its ligands.^5^, ^6^, ^25^ Preclinical murine studies have corroborated this, with aPD‐1 observed to augment antitumor responses and delay metastasis in models of melanoma, colorectal and pancreatic cancer.^5^

In a phase‐I trial, nivolumab, an IgG4 antibody directed against PD‐1, produced partial responses in 17% NSCLC patients (*n* = 122), with durable disease stability.^39^ This was followed by a phase‐III clinical trial comparing nivolumab and docetaxel (chemotherapy) as a second line treatment for patients with advanced NSCLC, or other cancer.^40^ The objective response rate was 20% for nivolumab, compared to 9% with docetaxel.^40^ Following these results, The FDA approved the use of nivolumab in 2016 for advanced NSCLC.

Pembrolizumab, another IgG4 antibody directed against PD‐1, has also demonstrated impressive efficacy in NSCLC. In an open‐label phase‐III clinical trial, treatment‐naïve advanced NSCLC patients with greater than 50% PD‐L1 tumor cell expression (*n* = 305) demonstrated significantly longer progression‐free (10.3 months vs 6.0 months) and 6‐month overall survival (80.2% vs 72.4%), with fewer adverse events when treated with pembrolizumab compared to first‐line platinum‐based chemotherapies.^41^ This study led to the FDA approving pembrolizumab as first‐line treatment for patients with a tumor proportion score for PD‐L1 of 50% or greater.

### Adoptive cell transfer

Adoptive cell transfer is an exciting development in cancer immunotherapy that involves the transfusion of *in vitro* expanded and activated autologous lymphocytes to enhance the antitumor immune response. These isolated cells can also be genetically engineered, enabling introduction of TCRs with high tumor avidity, such as chimeric antigen receptor (CAR) T cells. ACT has proven to be highly effective for metastatic melanoma patients; however, its use in lung cancer remains novel.^2^

Adoptive transfer of cytokine‐induced killer (CIK) cells, a heterogeneous population of T cells with a NK cell phenotype (CD3^+^CD56^+^), represents perhaps the most thoroughly investigated form of ACT for lung cancer. In one recent study, Chen and colleagues found DC‐activated CIK cells in combination with standard platinum‐based doublet chemotherapy to be well tolerised and to significantly improve 3‐year survival compared to chemotherapy alone in NSCLC patients (50.7% vs 33.8%, *P* = 0.036, *n* = 68/group).^42^

Other forms of ACT have become an area of increased interest in lung oncology research. A randomised phase‐III clinical trial (*n* = 101) assessed the efficacy of DC‐activated CTL adoptive transfer combined with standard platinum‐based chemotherapy for post‐surgical NSCLC patients. The addition of ACT to chemotherapy led to a significant increase in 7‐year survival compared to the chemotherapy‐alone arm (55.1% vs 38.1%).^43^ Further studies are investigating expansion of autologous antitumor T central memory cells that exhibit long‐term survival and regeneration, and are in clinical trial in post‐operative chemotherapy‐treated NSCLC patients (NCT03402156). Similarly, patient *ex vivo* anti‐PD1 antibody‐stimulated TILs in combination with chemotherapy docetaxel and cisplatin regime are in progress in clinical trial (NCT03903887).

Finally, anti‐mucin CAR T‐cell therapy in lung cancer is currently in clinical trial in patients with advanced NSCLC (NCT03198052, NCT02587689).

### Neoantigens and vaccination

The host immune system is capable of recognising and targeting tumor cells. Numerous sources of TAAs and neoantigens arise due to mutation of oncogenes and suppressor genes, re‐expression of foetal proteins and oncogenic viral proteins, and/or overexpression of normal proteins.^2^, ^9^

In order to stimulate an antitumor immune response, neoantigens must be presented to T cells in the context of MHC molecules. To identify mutations, patient tumor samples are sequenced using next‐generation sequencing (NGS) technology for aberrations compared to their normal cellular DNA. Mutation expression is confirmed by RNA‐Seq and MHC binding potential determined *in silico*.^23^ Finally, neoantigen peptide is compared to the normal (wild type; WT) peptide to identify tumor‐specific T‐cell reactivity.^2^, ^7^

The ability to identify tumor‐specific neoantigens via NGS platforms has reinvigorated anticancer vaccination strategies.^44^ Patients can potentially be vaccinated with their own tumor‐specific neoantigens, representing a form of personalised medicine.^2,7,^ Current vaccination strategies combine chemotherapy and/or immunotherapy treatment with peptide to stimulate antitumor immunity.^2^

Currently, numerous neoantigen vaccination clinical trials are active and recruiting for NSCLC or SCLC (NCT03639714; NCT03715985), the outcome of which will guide future vaccination strategies.

## Novel immune checkpoint blockade targets for lung cancer

### CD40

Agonistic antibodies targeting CD40 (aCD40) aims to license DCs, upregulating costimulatory molecules required for cross‐presentation without CD4^+^ T‐cell help. Preclinical murine studies from our laboratory and others have demonstrated efficacy of aCD40 monotherapy against solid tumors, including mesothelioma;^45^ however, low tumor burden was required for regression.^19^, ^46^ This antitumor activity was largely ascribed to aCD40 driving dissemination of cross‐primed CTLS from TDLNs into the periphery.

In humans, a phase‐I clinical trial in various solid cancers observed partial responses in 14% (*n* = 29) of patients treated with aCD40 monotherapy. However, despite this, combination aCD40 therapy is demonstrating improved benefit. A phase‐I clinical trial evaluating dual chemotherapy (carboplatin/paclitaxel) + aCD40 (CP‐870,893) demonstrated 20% of advanced solid tumor patients (*n* = 30) to achieve a partial response.^47^ Additionally, combination of aCD40 with other immunotherapies has been observed to be effective. A phase‐I clinical trial combining aCD40 (CP‐870,893) + aCTLA‐4 (tremelimumab) observed 27.3% of metastatic melanoma patients (*n* = 24) to achieve an objective response, with two complete responses observed.^48^ Unfortunately, trials investigating aCD40 therapy in NSCLC are currently limited; however, clinical trials are ongoing for dual aCD40 (APX005M) + nivolumab in advanced NSCLC (NCT03123783).

### OX40

OX40 (CD134) and its ligand, OX40L (CD252), are expressed transiently on activated T‐cell surfaces and constitutively on APCs, respectively. Ligation of OX40 provides costimulation to activated T cells, increasing their survival and prolonging responses.^49^ Studies have observed Treg OX40 ligation to promote accumulation of quiescent Tregs and antagonise FOXP3 induction in naïve CD4^+^ T cells.^49^ Therefore, therapeutically targeting the OX40 axis to stimulate long‐term T‐cell responses and reduce Treg burden is an attractive target for treatment of lung cancers. Preclinical murine tumor models of mammary carcinoma have demonstrated tumor regression with aOX40 + aPD‐1 dual treatment, with efficacy likely dependent on augmentation to CD4^+^ T‐cell helper functions, enhancing CD8^+^ T‐cell cross‐priming and recruitment to TDLNs.^50^

Clinical trials have validated anti‐OX40 efficacy. A phase‐I trial in advanced solid cancer patients reported durable antitumor responses following aOX40 monotherapy, with at least one metastatic nodule regressing in 40% (*n* = 30) of patients.^51^ Despite the promising responses observed in aOX40 monotherapy, combination therapy will likely improve efficacy further. Studies from our laboratory have demonstrated combination aOX40 + aCTLA‐4 therapy to improve survival and tumor regression in preclinical mesothelioma models.^52^ Unfortunately, there are limited data on aOX40 therapy in a lung cancer setting, although clinical trials are ongoing for NSCLC (NCT02315066).

## Combination immunotherapy

Despite the promise that many immunotherapies are showing, not all patients respond.^5^, ^6^ Thus, many research efforts have now focused on combining immunotherapies, with data indicating the potential for synergism between therapies.

Promising results with ICPB monotherapy have been followed up by preclinical data suggesting that aPD‐1 antibody in combination with aCTLA‐4 antibody may increase antitumor activity.^52^ In clinical trials, nivolumab and ipilimumab combination therapy achieved an overall response rate of 43% in unselected patients with NSCLC, compared with 23% in the nivolumab monotherapy group; and in the PD‐L1 positive subgroup, nivolumab in combination with ipilimumab showed a response rate of 57%, whilst nivolumab alone was 28% (CheckMate 012 study).^53^ Further, a phase‐II clinical trial investigating dual ipilimumab + nivolumab therapy demonstrated enhanced and durable antitumor responses, with a 30% overall objective response rate in advanced NSCLC patients (*n* = 252; CheckMate 568).^54^ However, adverse events of grade 3 or above of combination therapy reached 37%, thereby limiting the widespread clinical application. Interestingly, patients with a higher tumor PD‐L1 expression had improved responses. As such, it may be that improved predictive frameworks can better predict those patients that will respond to treatment.

Most recently, given ipilimumab is effective in the initiation of the immune response current clinical trials have staggered application of antibody therapy, with ipilimumab for initiation of an immune response followed by PD‐1 blockade (phase I; NCT03527251). Other recruiting clinical trials include the ICOS inhibitor vopratelimab in combination with ipilimumab in patients with advanced and/or refractory NSCLC (NCT0398936) after prior aPD‐1/anti‐PD‐L1 therapy.

Other immune therapy research has similarly focused on combination therapy, with anti‐PD‐1/TIM‐3 bi‐specific antibodies in clinical trial for solid tumors, including NSCLC (NCT03708328).

## ICPB therapy in combination with chemotherapy and radiotherapy

The somewhat recent findings indicating the immunomodulatory capacity of chemotherapy and radiotherapy have led to many studies to investigating their potential synergistic effects with immunotherapy in the treatment of NSCLC.

A recent randomised, double‐blinded, phase‐III trial of ipilimumab combined with platinum‐based chemotherapy (carboplatin and paclitaxel) for advanced NSCLC patients (*n* = 956) observed no additional survival benefit compared to chemotherapy alone.^55^ More promisingly, a double‐blinded phase‐III trial investigated the addition of pembrolizumab to chemotherapy (pemetrexed and a platinum‐based chemotherapy viz. cisplatin or carboplatin) in treatment‐naïve advanced NSCLC patients (*n* = 616; KEYNOTE‐189, NCT02578680). Here, the addition of pembrolizumab to chemotherapy resulted in significantly longer progression‐free survival (8.8 months vs 4.9 months) and overall survival at 12 months (69.2% vs 49.4%).^56^ The clinical standard platinum‐based doublet chemotherapy is now in clinical trial with combinations of ipilimumab and pembrolizumab (NCT03515629). Other chemotherapy immune checkpoint blockade combinations in clinical trial include avelumab (anti‐PD‐L1) with pemetrexed/carboplatin or gemcitabine/cisplatin treatment protocols (NCT03317496). Clinical trials are also investigating pembrolizumab as a first‐line treatment alone or in combination with pemetrexed carboplatin (NCT03793179). Further results are awaited.

Increasing evidence from both clinical and preclinical settings suggests that radiotherapy may be a useful partner for ICPB, causing beneficial immune modulation and release of TAAs but without the systemic toxicities associated with chemotherapy.^2^ Radiotherapy to enhance immunotherapy is currently clinical trials that are underway. These include anti‐CTLA‐4 and/or anti‐PD1 with fractionated radiotherapy (NCT03509584), radical dose image‐guided radiation therapy or ablative radiotherapy with standard immunotherapy treatment (NCT03176173; NCT03110978). More complex phase‐II clinical trials are pending for combination immunotherapy (anti‐CTLA‐4) and chemotherapy (carboplatin/paclitaxel) with surgery and/or radiotherapy (NCT03965468).

The outcomes of these immunotherapy combination chemotherapy/radiation therapy will guide the future of lung cancer clinical practice.

## Immunotherapy and surgery

Surgery remains the front‐line primary curative treatment for localised solid malignancies, including lung cancer. However, the overall effect of tumor debulking surgery on the antitumor immune response remains contentious. It is suggested that surgery itself may suppress systemic immunity, abrogating CTL function, and thus promoting metastatic outgrowth.^57^ Conversely, surgical removal of tumor may alleviate immunosuppression, providing a more amenable environment for immunotherapy against metastatic or residual disease.^46^ New studies are determining the impact of neoadjuvant immunotherapy in stimulating a T‐cell immune response to tumor followed by surgical removal of tumor along with the associated immunosuppressive environment may improve patient outcomes. However, LN resection for staging purposes may impact on immunotherapy outcomes.

Phase‐III clinical trial recruitment for post‐surgical resection and chemotherapy treatment NSCLC patients, for follow‐up treatment with nivolumab, is in progress (NCT02595944). In the neoadjuvant setting, a recent clinical trial for nivolumab, in resectable NSCLC patients, determined a major pathological response in 45% of patients (*n* = 21).^58^ Further, they also observed expansion of previously undetectable neoantigen‐specific T cells, perhaps suggestive of priming of *de novo* immune responses.

During surgery, tumor‐free TDLNs are frequently removed for staging purposes, removing the ‘factory’ of T‐cell immune stimulation.^59^ A preclinical study in a murine model of colorectal cancer observed TDLN resection to reduce PD‐1 blockade efficacy, likely due to failure of adequate T‐cell cross‐priming.^60^ There are limited clinical data on the impact of lymphadenectomy on the post‐surgical immunotherapy response; as such, caution should be used in the clinical integration of neoadjuvant or adjuvant immunotherapy with surgery.

## Immunotherapy in combination with other treatments

Most recently, it has become evident that effective antitumor responses to immunotherapy may be dependent on the TME prior to treatment.^61^. Accordingly, new exciting clinical trials are emerging to modulate TME prior to or at the time of immunotherapy treatment. Production of immunosuppressive kynurenine by tumor cells is limited by inhibitor of indoleamine 2,3‐dioxygenase 1 (IDO1; BMS‐986205) and is currently in phase‐I clinical trial in combination with nivolumab alone or in combination with ipilimumab (NCT02658890). Other tumor treatments such as plinabulin targeting DC maturation are being investigated in combination with nivolumab and ipilimumab for objective response in SCLC (NCT03575793). Additionally, other clinical trials in lung cancer include treatment combination with anti‐PD1/anti‐PD‐L1 and/or anti‐CTLA‐4 therapy with inhibition of G protein‐coupled receptors (PBF509; NCT02403193); activation of CD122 for T‐cell expansion (NKTR‐214 cytokine; NCT02983045); and receptor tyrosine kinase inhibitors (nintedanib; NCT03377023).

## Conclusion

Improved understanding of tumor‐immune interactions and the role of T cells in lung malignancies have undermined the classical notion of lung cancer being a non‐immunogenic disease. Expanding knowledge has driven development of novel immunotherapeutic approaches, such as immune checkpoint blockade therapy, which has demonstrated remarkable clinical success and revolutionised advanced lung cancer treatment. Further investigation into the combination of these immunotherapies with other immunotherapies or conventional therapies is a current area of concerted investigation. Adjuvant immune checkpoint blockade therapy may reduce post‐surgical recurrences, yet little is understood about the impact tumor‐draining LN resection at the time of surgery. Elucidation of the location of action and the specific immune‐modulating mechanisms governing the efficacy of immune checkpoint blockade will improve strategic combination therapy to improve patient outcomes.

## Conflict of interest

The authors declare no conflict of interest.
